# Investigation of Copy Number Variations (CNVs) of the Goat *PPP3CA* Gene and Their Effect on Litter Size and Semen Quality

**DOI:** 10.3390/ani12040445

**Published:** 2022-02-12

**Authors:** Yangyang Bai, Taiyuan Zhang, Nuan Liu, Congliang Wang, Zhengang Guo, Chuanying Pan, Haijing Zhu, Xianyong Lan

**Affiliations:** 1 Key Laboratory of Animal Genetics, College of Animal Science and Technology, Breeding and Reproduction of Shaanxi Province, Northwest A&F University, Yangling 712100, China; bai345@126.com (Y.B.); zhangtaiyuan666@163.com (T.Z.); liunuan_1115@126.com (N.L.); gzg0857@163.com (Z.G.); panyu1980@126.com (C.P.); 2 Shaanxi Provincial Engineering and Technology Research Center of Cashmere Goats, Yulin University, Yulin 719000, China; wcl15596064203@126.com (C.W.); haijingzhu@yulinu.edu.cn (H.Z.); 3 Life Science Research Center, Yulin University, Yulin 719000, China; 4 Bijie Animal Husbandry and Veterinary Science Research Institute, Bijie 551700, China

**Keywords:** goat, *PPP3CA*, copy number variation (CNV), litter size, semen quality

## Abstract

**Simple Summary:**

*PPP3CA* is one of the candidate genes for goat reproduction, but no studies have been carried out yet. Therefore, the purpose of this study was to determine the associations between copy number variations in the goat *PPP3CA* gene and litter size and semen quality in goats, including Shaanbei white cashmere goats (SBWC) (*n* = 353) and Guizhou Heima (GZHM) goats (*n* = 64). Based on the association analysis, the results showed that only CNV1 (copy number variation 1) and CNV2 (copy number variation 2) were distinctly related to the first-birth litter size in female goats (*p* = 7.6802 × 10^−11^; *p* = 5.0895 × 10^−9^), and they were also significantly associated with the semen quality of SBWC goats (*p* < 0.05). These findings prove that the *PPP3CA* gene plays an important role in reproduction traits in goats.

**Abstract:**

Copy number variations (CNVs) have many forms of variation structure, and they play an important role in the research of variety diversity, biological evolution and disease correlation. Since CNVs have a greater impact on gene regulation and expression, more studies are being finalized on CNVs in important livestock and poultry species. The *protein phosphatase 3 catalytic subunit alpha* (*PPP3CA*) is a key candidate gene involved in the goat fecundity trait, and has important effects on precocious puberty, estrogen signal transduction pathways and oocyte meiosis. Additionally, *PPP3CA* also has a dephosphorylation effect in the process of spermatogonial stem cell meiosis and spermatogenesis. So far, there is no research on the relationship between the copy number variations of the *PPP3CA* gene and reproduction traits. Therefore, the purpose of this study was to determine the association between copy number variations in the goat *PPP3CA* gene and litter size and semen quality in Shaanbei white cashmere goats (SBWC) (*n* = 353) and Guizhou Heima goats (*n* = 64). Based on the association analysis, the results showed that only CNV1 and CNV2 within the *PPP3CA* gene were distinctly related to the first-birth litter size in female goats (*p* = 7.6802 × 10^−11^; *p* = 5.0895 × 10^−9^, respectively) and they were also significantly associated with the semen quality of SBWC goats (*p* < 0.05). In addition, individuals with Loss genotypes demonstrated better phenotypic performance compared to those with other types. Therefore, CNV1 and CNV2 of the *PPP3CA* gene are potentially useful for breeding, as they are linked to important goat reproduction traits.

## 1. Introduction

Located on chromosome 6, the protein phosphatase 3 catalytic subunit alpha (*PPP3CA*), was first discovered in brain tissue in 1979, and has a variety of biological functions [1]. To date, the main study of this gene was related to cell growth and development, immune response, neurodevelopmental diseases and spermatogenesis [2,3,4]. Hence, *PPP3CA* was suggested to be a key candidate gene involved in livestock animals’ reproductive traits [5]. For example, Dias et al. (2015) found that the *PPP3CA* gene had a crucial effect on precocious puberty [6]. Additionally, a whole-genome selective scanning analysis in 31 Dazu black goats found that the *PPP3CA* gene was annotated to reproduction-related pathways [7], which had an important influence on the estrogen signal transduction pathway and oocyte meiosis [6]. Previously, Wan et al. (2014) found that the *PPP3CA* gene played a key role in the control of skeletal muscle fibers, and that it was expressed in various muscle tissues [8]. It can inhibit the expression of *fibroblast growth factor 23 (FGF23)* and participate in the regulation of skeletal muscle growth and development [9]. In our previous study, a 20 bp indel mutation in the *PPP3CA* gene affected goat litter size through the differential binding of the reproduction-related transcription factor NF-1 [10]. Several studies had demonstrated that *PPP3CA* is a potential phosphatase in mouse testes, which may regulate the function of dynein 2 (DNM2) in the acrosome of mature cells, thereby affecting spermatogenesis. At the same time, the inactivation of *PPP3CA* not only inhibited mouse testis development, but also reduced the number of mature sperm, indicating that this gene plays a vital role in sperm production and testicular development [11,12,13]. Therefore, we hypothesized that *PPP3CA* might affect litter size and semen quality traits in goats, and could be an effective DNA maker than can be applied in MAS breeding.

Copy number variation (CNV) is the main form of genome structural variation and is an important source of genetic variation and phenotypic differences. Therefore, it plays an essential role in the occurrence and development of complex traits [14,15,16]. In 2004, Sebat et al. studied the CNV of the human genome and found that it was involved in the regulation of neural function, cell growth, metabolism and disease [17]. In terms of the number of mutated nucleotides and the impact on the individual traits of organisms, CNV may be more important for the genetic variation and diversity of organisms than SNPs and InDels. Since CNV has a greater impact on gene regulation and expression, more studies are being conducted on CNV in important livestock and poultry species [18,19,20]. As a critical reproductive trait, litter size and semen quality affect the economic benefits of the goat industry. The average lambing rate of SBWC goats is about 109% and, therefore, it still needs to be improved [21,22]. Consequently, we hope to discover the major genes that affect reproduction in goats. In our previous study, Bi et al. (2021) found five potential copy number variations within the main fecundity Fec ^B^ gene, and two of these CNVs could significantly affect goat litter size. It was also found that two InDel loci in the *down syndrome cell adhesion molecule like 1 (DSCAML1)* gene were related to the semen quality and litter size of goats [23,24].

Here, we selected 353 Shaanbei white cashmere goats (female: *n* = 307; male: *n* = 46) from Shaanxi province and Guizhou Heima goats (GZHM) (*n* = 64) from Guizhou province as experimental samples to analyze the association between copy number variations of the *PPP3CA* gene and litter size and semen quality to provide a scientific basis for the application of MAS breeding in the goat breeding industry, and thereby improve reproductive traits.

## 2. Materials and Methods

All experiment procedures were approved by the Faculty Animal Policy and Welfare Committee of Northwest A&F University (FAPWC-NWAFU) and experiments on animals were completely in agreement with the local animal welfare laws and policies (NWAFU-314020038).

### 2.1. Sample Collection and DNA Isolation

To study the effect of the *PPP3CA* gene copy number variations in SBWC goats, 353 adult goats (2–3 years old, female goats (*n* = 307) and male goats (*n* = 46)) were collected in Shaanxi province, China. In order to study the distribution of the three copy number variations of the *PPP3CA* gene in another goat breed, we randomly choose Guizhou Heima (GZHM) goats as a control. Hence, we also collected ear samples of adult female GZHM goats (*n* = 64) in Guizhou province. The Shaanbei white cashmere (SBWC) goats were kept under standard conditions, including the same diet and feeding and management conditions. According to the full pedigree recorded on the farm, the individuals we selected are not related to each other. This means that all individuals of 3–4 generations do not share a common ancestor [25]. In addition, the agricultural technical station provided the first-birth litter size records of female goat (*n* = 307). All genomic DNA was isolated from ear tissue from 417 samples according to the phenol–chloroform method [24,25]. We use the artificial vagina method to ejaculate the trained ram before semen collection to promote the production of fresh semen, ensure the quality of semen and improve the libido of the breeding ram. Finally, we recorded the amount of ejaculate [26].

### 2.2. Detection of Various Indicators of Semen Quality

#### 2.2.1. The Determination of Semen Concentration

The semen was diluted at a ratio of 1:100 and counted using a hemocytometer. The sperm count was observed in the five squares on the hemocytometer under a microscope, and this allowed us to calculate the sperm concentration [26].

#### 2.2.2. The Determination of the Percentage of Viable Sperm

Semen samples were diluted 100 times with 0.9% sodium chloride solution, then mixed evenly, and dropped onto a glass slide on a constant temperature table (adjust the temperature to 37–38 °C) and covered with a clean cover glass. Next, we examined the number of sperm in a linear motion under the microscope and calculated the sperm motility [27].

#### 2.2.3. The Determination of Sperm Deformity Rate

The examination of the sperm deformity rate involved smearing the semen so that it could be observed directly under the microscope, and then staining it with common staining solution (methylene blue) or red and blue ink. Then, we washed it with water and dried it for microscopic examination. We examined at least 200 sperm cells, and the percentage of deformity was calculated [28].

#### 2.2.4. The Determination of Sperm Plasma Membrane Integrity

The integrity of the sperm plasma membrane was detected using the HOST hypotonic swelling method. The centrifuge tube and permeate were preheated, 10 μL of semen was added to 100 μL hypotonic solution, and the cells were incubated in a water bath at 37 °C for 30 min. Spermatozoa were observed under a microscope. Sperm with bent tails are sperm with the intact plasma membrane, while sperm with uncurved tails are sperm with an incomplete plasma membrane. We counted 200 spermatozoa in a certain field of view, repeated the count twice, and calculated the average percentage of swollen spermatozoa [29].

### 2.3. Primer Design and CNV Detection

In this study, we used the Animal Omics Database (http://animal.nwsuaf.edu.cn/ accessed on 10 September 2021) to search for CNVs of the goat *PPP3CA* gene (NC_030813.1) and found that there are three CNV mutations in the introns of the *PPP3CA* gene (Table 1) [30]. The quantitative PCR primer pairs (CNV1, CNV2 and CNV3) were used to detect the copy number variations of the *PPP3CA* gene in different individuals, and they were designed using Primer Blast (https://www.ncbi.nlm.nih.gov/tools/primer-blast/ accessed on 12 September 2021). Following a previous study, the MC1R gene was used as internal control [20]. Additionally, all primers’ pair sequence information is shown in Table 2. The quantitative real-time polymerase chain reaction (qPCR) amplification reaction system volume (10 μL) and the procedure are as described in our previous studies [23,31].

### 2.4. Copy Number Analysis and Statistical Analyses

First, we used CNVcaller software to analyze the resequencing results of the database and identify the position of repeated breakpoints in the *PPP3CA* gene region in goats [32]. Then, we amplified each sample via qPCR with three replicates for each pair of primers. Subsequently, the qPCR results of the copy number of the *PPP3CA* gene were analyzed using the 2*2^−^^∆CT^ method, where ∆CT = Ct_target_ − Ct_internal_, Ct_target_ is the number of cycles when the target gene amplification reaches the threshold and Ct_internal_ is the cycle number when the internal reference gene amplification reaches the threshold. The CNVs were divided into three types, Gain (2 × 2^−^^∆CT^ ≥ 3), Loss (2 × 2^−^^∆CT^ < 2), and Medium (2 × 2^−^^∆CT^ = 2) [20,23].

The following model was used for the association analysis of CNV types and litter size and semen quality: Y_ijk_ = µ + S_i_ + G_l_ + e_ijk_, where Y_ijk_ was the trait measured for each animal, µ was the overall population mean, S_i_ was the effect of age, G_l_ was the copy number variation type of each point and e_ijk_ was the random error [33]. A one-way ANOVA was used to analyze the association between litter size, semen quality and CNV types in the SPSS software.

## 3. Results

### 3.1. qPCR Primer Detection

**Figure 1:**
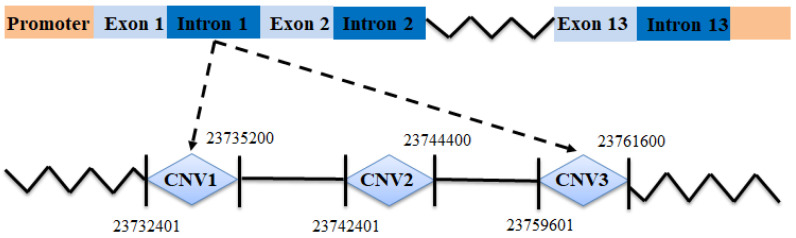
Gene structure of the goat *PPP3CA* gene.

**Figure 2:**
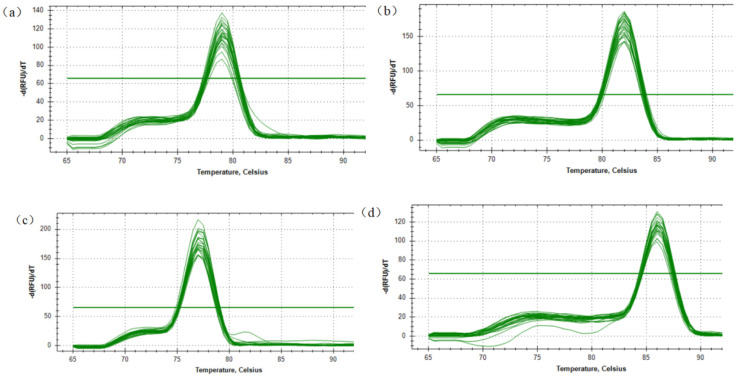
Melting curve of CNV1 (**a**), CNV2 (**b**) and CNV3 (**c**) sites of the *PPP3CA* and *MC1R* (**d**) genes.

Three CNVs within the goat *PPP3CA*, namely, CNV1 (NC_030813.1 g.23732401–23735200, intron), CNV2 (NC_030813.1 g.23742401–23744400, intron) and CNV3 (NC_030813.1 g.23759601-23761600, intron), were detected in SBWC goats (*n* = 353 and GZHM goats (n = 64) (Table 1 and Table 2). Additionally, the gene structure of the goat *PPP3CA* gene was drawn as shown in Figure 1. The melting curves of the three CNV mutant primers of the *PPP3CA* gene and the *MC1R* gene are shown in Figure 2. It shows that the dissolution peak patterns of the *PPP3CA* gene and the internal reference gene *MC1R* gene are single, and the dissolution temperature is above 80 °C, indicating that the quantitative primers belong to a specific amplification and have a good amplification efficiency.

### 3.2. Distribution of Different CNV Types in Goats

In order to determine the distribution of the *PPP3CA* gene copy number in SBWC goats, we chose 307 female goats and 46 male goats, and 64 female GZHM goats. According to the 2 × 2^−^^∆CT^ method, we divided the CNV types into three classes, including Loss type (0~2), Medium type (2) and Gain type (>2). As shown in Table 3, CNV1 and CNV2 displayed three types (Loss, Medium and Gain) in both male and female SBWC goats, while CNV3 had only one type (Gain) in male goats. In addition, in order to determine the distribution of copy number variations of the *PPP3CA* gene in different goat breeds, we detected the copy number of the PPP3CA gene in GZHM goats. The results showed that CNVs within the *PPP3CA* gene displayed different distributions in two populations.

### 3.3. Association Analysis of CNV Type and Goat Litter Size

Since no reproductive trait records were available in GZHM goats, we only used association analysis to explore the relationship between different CNV types and litter size in 307 female SBWC goats. The statistical analyses showed that CNV1 and CNV2 were significantly associated with goat litter size (*p* = 7.6802 × 10^−11^; *p* = 5.0895 × 10^−9^, respectively). For CNV1, the Loss genotype had the higher litter size compared to the Gain and Medium genotypes. And for CNV2, the Medium genotype had the highest litter size compared to the Gain and Loss genotypes. Meanwhile, there was no significant difference between CNV3 and litter size (*p* = 0.487) (Table 4).

### 3.4. Association Analysis of CNV Type and Goat Semen Quality

Herein, we examined the association of the *PPP3CA* gene copy number variations with the corresponding individual semen quality in 46 Shaanbei white cashmere goats (male) using the one-way ANOVA method. As shown in Table 5, CNV1 was correlated with ejaculation volume (EV; *p* = 0.035), semen concentration (SC; *p* = 0.048), sperm viability (SV; *p* = 0.026) and live sperm count (LS; *p* = 2.3 × 10^−4^). Moreover, CNV2 was significantly correlated with semen concentration (SC; *p* = 0.030) and live sperm count (LS; *p* = 0.005) (Table 5).

## 4. Discussion

As one of the most important economic traits in large-scale breeding, goat reproductive traits account for a large proportion of the overall income of breeders. However, the low heritability of reproductive traits, the long cycle, the slow breeding progress and the complex relationship with growth traits are the main reasons for the economic disadvantages of goat breeding [34,35]. With the development of biotechnology, marker-assisted selection (MAS) has been widely used in goat breeding to rapidly enhance goat reproductive performance. Several natural genetic variations, including SNPs, InDels and CNVs, have been identified. As an important source of genome structural variation, CNV has a large mutation segment, which causes huge genetic effects on individuals, thus providing a molecular basis for the study of important economic traits of livestock and poultry. Currently, researchers are conducting classification studies based on copy numbers by identifying the differences in the time threshold number in the qPCR cycle. In this study, the intention was to explore the effects of three CNVs in the *PPP3CA* gene on litter size and semen quality in goats.

Protein phosphatase 3 (PPP3), also known as calcineurin (calcineurin, CaN), is the only Ser/threonine regulated by Ca2 +/calmodulin (CaM) [36]. The protein phosphatase, which is widely present in a variety of eukaryotic cells, plays a key role in the cellular response mediated by the Ca2+/CaN/NFAT signaling pathway [37]. The *PPP3CA* gene can play a role in spermatogonial stem cell meiosis and spermatogenesis through dephosphorylation, thereby affecting male reproduction [13]. At present, there are relatively few studies on the effect of this gene on reproductive traits. Bai et al. (2021) found that the 20 bp indel polymorphism may affect the litter size by binding to the reproduction-related transcription factor NF-1 in goats [10]. Based on these findings, we speculated that *PPP3CA* is a candidate gene for reproductive traits in goats.

On the basis of the Animal Omics Database (http://animal.nwsuaf.edu.cn/ accessed on 8 September 2021) built by the Jiangyu team of our college (College of Animal Science and Technology, Northwest A&F University), the CNVcaller software was used to analyze the existing whole-genome resequencing results. Additionally, the results found that there are breakpoints in this group of Shaanbei white cashmere (SBWC) goats. Therefore, the DNA sequencing results and bioinformation analysis proved that the CNVs within the *PPP3CA* gene region in SBWC are available, and there are several variable changes in CNV region. Additionally, the same CNV regions within this gene were also analyzed in other goat breeds using whole-genome resequencing and bioinformatic analysis (Supplementary Table S1). Since the whole-genome re-sequencing method and bioinformatic analysis are expensive and time-consuming, in this study, we detected the potential CNVs in big sample size of goats (*n* > 400) using the qPCR method, which is efficient and time-saving.

Herein, we examined the potential CNVs of the *PPP3CA* gene in SBWC female and male goats (*n* = 353) and the female GZHM goats (*n* = 64) for the first time. We found that among the three CNVs, CNV1 and CNV2 were significantly associated with first-born litter size (*p* < 0.01). For CNV1 and CNV2, individuals with Loss and Medium genotypes had advantages in goat litter size, respectively. In the male group (*n* = 46), we also detected the copy number variations of the *PPP3CA* gene. CNV1 and CNV2 displayed three types (Gain, Medium and Loss), while CNV3 had only one type (Gain). This phenomenon may be due to the difference between our selected groups and the intensity of artificial selection. To analyze the associations between CNVs and semen quality, we recorded 46 male goats’ ejaculate volume, sperm concentration, sperm motility, sperm deformity rate, plasma membrane integrity rate, live sperm count and other indicators to measure semen quality. The results showed that CNV1 was correlated with ejaculation volume (EV; *p* = 0.035), semen concentration (SC; *p* = 0.048), sperm viability (SV; *p* = 0.026) and live sperm count (LS; *p* = 2.3 × 10^−4^). Moreover, CNV2 was significantly correlated with semen concentration (SC; *p* = 0.030) and live sperm count (LS; *p* = 0.005). The individuals with the Loss copy number had more SC and LS than individuals with the other two types, suggesting that Loss-type individuals performed better than the other two individuals in terms of semen quality. Although China has abundant resources for goat breeding, poor fecundity restricts the development of the goat industry. From this perspective, CVN1 and CNV2 may be suitable for further selection and breeding. The role of the *PPP3CA* gene in goat reproduction, as well as a series of signal transduction and regulation mechanisms in vivo, is still unclear, so further research is needed.

It is known that cAMP acts through protein kinases (PKA), and the absence of cAMP or PKA leads to insufficient sperm motility and male infertility [38]. *PPP3CA* is one of the genes in the PKA pathway, and the variation in its intron region may affect the combination ability of DNA sequence and transcription factors, thereby indirectly affecting the litter size and semen quality of goats. So far, some introns contain transcriptional regulatory sequences that bind transcriptional regulators to control the rate of transcription. Furthermore, changes in the intron sequence may also affect splicing patterns and, thus, affect phenotype [39]. For example, Kang et al. (2019) found that mutations in the intron region of the goat *MARCH1* gene regulate ovarian expression levels and affect the litter size [40]. However, the underlying molecular mechanism of CNV1 and CNV2 of the *PPP3CA* gene on the litter size and semen quality of goats requires further investigation.

## 5. Conclusions

Based on the association analysis, the results showed that only CNV1 (copy number variation 1) and CNV2 (copy number variation 2) were distinctly related to the first-birth litter size in female goats (*p* = 7.6802 × 10^−11^; *p* = 5.0895 × 10^−^^9^, respectively) and were also significantly associated with the semen quality of SBWC goats (*p* < 0.05). In addition, individuals with Loss genotypes demonstrated better phenotypic performance compared to those with other types. Therefore, CNV1 and CNV2 of the *PPP3CA* gene are potentially useful for breeding, as they are linked to important goat reproduction traits.

## Figures and Tables

**Table 1:** The CNV loci of the goat *PPP3CA* gene.

Primers	Start	End	Length	Location	Reference Sequence
CNV1	23732401	23735200	2799	intron	NC_030813.1
CNV2	23742401	23744400	1999	intron	NC_030813.1
CNV3	23759601	23761600	1999	intron	NC_030813.1

**Table 2:** Primers used for detection of the CNV mutations and the relative expression of *PPP3CA*.

Primers	Sequences (5′-3′)	Sizes (bp)
CNV1	F: CCTCTGCCTACTCTTTCCTGC	106
	R: GGGGGAAATGGTCCCCAAA	
CNV2	F: AGAGGGAACCTCCATGGTCA	108
	R: CCAAGCCTCAACTCACTCCA	
CNV3	F: CCGACTCCCCCTGAAGTAGT	199
	R: TCTCCAGCAATGTTAGGCTGT	
MC1R	F: GGCCTGAGAGGGGAATCACA	126
	R: AGTGGGTCTCTGGATGGAGG	

**Table 3:** Typical frequencies of copy number variations within the *PPP3CA* gene in different goat breeds.

Breed	Loci	Sizes	Typic Frequencies		
			Gain	Medium	Loss
SBWC goat (female)	CNV1	300	0.363 (*n* = 109)	0.187 (*n* = 56)	0.450 (*n* = 135)
	CNV2	307	0.861 (*n* = 206)	0.153 (*n* = 47)	0.176 (*n* = 54)
	CNV3	301	0.781 (*n* = 235)	0.056 (*n* = 17)	0.163 (*n* = 49)
SBWC goat (male)	CNV1	46	0.370 (*n* = 17)	0.522 (*n* = 24)	0.108 (*n* = 5)
	CNV2	46	0.783 (*n* = 36)	0.130 (*n* = 6)	0.087 (*n* = 4)
	CNV3	46	1.000 (*n* = 46)	-	-
GZHM goat (female)	CNV1	64	0.047 (*n* = 3)	0.734 (*n* = 47)	0.219 (*n* = 14)
	CNV2	64	0.094 (*n* = 6)	0.703 (*n* = 45)	0.203 (*n* = 13)
	CNV3	64	0.875 (*n* = 56)	0.109 (*n* = 7)	0.016 (*n* = 1)

**Table 4:** Statistical association analysis of three CNVs of PPP3CA with litter sizes in goats.

Mutations	Sizes	Gain	Medium	Loss	*p*-Values
CNV1	300	1.40 ^B^ ± 0.05 (*n* = 109)	1.25 ^B^ ± 0.06 (*n* = 56)	1.72 ^A^ ± 0.04 (*n* = 135)	7.6802 × 10^−11^
CNV2	307	1.40 ^B^ ± 0.03 (*n* = 206)	1.79 ^A^ ± 0.06 (*n* = 47)	1.74 ^A^ ± 0.06 (*n* = 54)	5.0895 × 10^−9^
CNV3	301	1.50 ± 0.03 (*n* = 235)	1.41 ± 0.12 (*n* = 17)	1.57 ± 0.07 (*n* = 49)	0.487

Note: Values with different letters (A, B) within the same row differ significantly at (*p* < 0.01); data represented as means ± standard error.

**Table 5:** Statistical association analysis of two CNVs of *PPP3CA* with semen quality in goats.

Mutations	Parameters		Genotypes		*p*-Values
		Gain	Medium	Loss	
CNV1	EV	0.94 ± 0.09 ^ab^ (*n* = 17)	0.75 ^b^ ± 0.05 (*n* = 24)	1.12 ^a^ ± 0.18 (*n* = 5)	0.035
	SC	292.94 ^b^ ± 3.46 (*n* = 17)	271.10 ^b^ ± 3.37 (*n* = 24)	465.20 ^a^ ± 6.03 (*n* = 5)	0.047
	SV	50.90 ^B^ ± 0.03 (*n* = 17)	54.60 ^b^ ± 0.04 (*n* = 24)	74.00 ^Aa^ ± 0.04 (*n* = 5)	0.026
	SdR	34.00 ± 0.04 (*n* = 17)	27.50 ± 0.04 (*n* = 24)	21.10 ± 0.05 (*n* = 5)	0.320
	SaR	82.90 ± 0.02 (*n* = 15)	91.60 ± 0.01 (*n* = 22)	92.40 ± 0.03 (*n* = 5)	0.169
	LS	151.30 ^B^ ± 2.18 (*n* = 17)	147.20 ^B^ ± 1.77 (*n* = 24)	343.30 ^A^ ± 5.01 (*n* = 5)	2.3 × 10^−4^
CNV2	EV	0.82 ± 0.57 (*n* = 36)	1.00 ± 0.12 (*n* = 6)	0.83 ± 0.14 (*n* = 4)	0.141
	SC	269.50 ^b^ ± 2.64 (*n* = 36)	373.10 ^ab^ ± 5.08 (*n* = 6)	468.10 ^a^ ± 6.41 (*n* = 4)	0.030
	SV	53.30 ± 0.27 (*n* = 36)	60.80 ± 0.07 (*n* = 6)	65.00 ± 0.12 (*n* = 4)	0.315
	SdR	31.30 ± 0.03 (*n* = 36)	19.90 ± 0.05 (*n* = 6)	24.80 ± 0.05 (*n* = 4)	0.343
	SPR	90.70 ± 0.02 (*n* = 33)	82.80 ± 0.03 (*n* = 5)	77.80 ± 0.12 (*n* = 4)	0.157
	LS	146.30 ^B^ ± 1.54 (*n* = 36)	215.80 ^AB^ ± 2.82 (*n* = 6)	314.10 ^A^ ± 5.14 (*n* = 4)	0.005

Note: EV, Ejaculation volume (mL); SC, Semen concentration(million/mL); SV, Sperm viability (%); SdR, Sperm deformity rate (%); SPR, Sperm plasma membrane integrity rate (%); LS, Live sperm count (million/mL). Values with different letters (a, b/A, B) within the same row differ significantly at (*p* < 0.05/*p* < 0.01); data represented as means ± standard error.

## Data Availability

Data are available upon request from corresponding author.

## Supplementary Materials

The following supporting information can be downloaded at: http://www.mdpi.com/article/10.3390/ani12040445/s1. Table S1: CNVcaller software analysis in different goat breeds.

## References

[1] Tash J.S., Krinks M., Patel J., Means R.L., Klee C.B., Means A.R. (1988). Identification, characterization, and functional correlation of calmodulin-dependent protein phosphatase in sperm. J. Cell Biol..

[2] Rydzanicz M., Wachowska M., Cook E.C., Lisowski P., Kuźniewska B., Szymańska K., Diecke S., Prigione A., Szczałuba K., Szybińska A. (2019). Novel calcineurin A (PPP3CA) variant associated with epilepsy, constitutive enzyme activation and downregulation of protein expression. Eur. J. Hum. Genet..

[3] Daei-Farshbaf N., Aflatoonian R., Amjadi F.S., Nikniyaz H., Taleahmad S., Bakhtiyari M. (2021). Identification of calcineurin as a predictor of oocyte quality and fertilization competence based on microarray data. Comput. Biol. Chem..

[4] Panneerselvam S., Wang J., Zhu W., Dai H., Pappas J.G., Rabin R., Low K.J., Rosenfeld J.A., Emrick L., Xiao R. (2021). PPP3CA truncating variants clustered in the regulatory domain cause early-onset refractory epilepsy. Clin Genet..

[5] Islam R., Liu X., Gebreselassie G., Abied A., Ma Q., Ma Y. (2020). Genome-wide association analysis reveals the genetic locus for high reproduction trait in Chinese Arbas cashmere goat. Genes Genom..

[6] Dias M.M., Souza F.R., Takada L., Feitosa F.L., Costa R.B., Diaz I.D., Cardoso D.F., Tonussi R.L., Baldi F., Albuquerque L.G. (2015). Study of lipid metabolism-related genes as candidate genes of sexual precocity in Nellore cattle. Genet. Mol. Res..

[7] E G.X., Zhao Y.J., Huang Y.F. (2019). Selection signatures of litter size in Dazu black goats based on a whole genome sequencing mixed pools strategy. Mol. Biol. Rep..

[8] Wan L., Ma J., Xu G., Wang D., Wang N. (2014). Molecular cloning, structural analysis and tissue expression of protein phosphatase 3 catalytic subunit alpha isoform (PPP3CA) gene in Tianfu goat muscle. Int. J. Mol. Sci..

[9] Bär L., Großmann C., Gekle M., Föller M. (2017). Calcineurin inhibitors regulate fibroblast growth factor 23 (FGF23) synthesis. Naunyn Schmiedebergs Arch. Pharmacol..

[10] Bai Y., Li J., Zhu H., Liu J., Dong S., Li L., Qu L., Chen H., Song X., Lan X. (2021). Deletion mutation within the goat PPP3CA gene identified by GWAS significantly affects litter size. Reprod. Fertil. Dev..

[11] Martin L.J., Chen H., Liao X., Allayee H., Shih D.M., Lee G.S., Hovland D.N., Robbins W.A., Carnes K., Hess R.A. (2007). FK506, a calcineurin inhibitor, prevents cadmium-induced testicular toxicity in mice. Toxicol. Sci..

[12] Reid A.T., Lord T., Stanger S.J., Roman S.D., McCluskey A., Robinson P.J., Aitken R.J., Nixon B. (2012). Dynamin regulates specific membrane fusion events necessary for acrosomal exocytosis in mouse spermatozoa. J. Biol. Chem..

[13] Redgrove K.A., Bernstein I.R., Pye V.J., Mihalas B.P., Sutherland J.M., Nixon B., McCluskey A., Robinson P.J., Holt J.E., McLaughlin E.A. (2016). Dynamin 2 is essential for mammalian spermatogenesis. Sci. Rep..

[14] Di Gerlando R., Mastrangelo S., Moscarelli A., Tolone M., Sutera A.M., Portolano B., Sardina M.T. (2020). Genomic structural diversity in local goats: Analysis of copy-number variations. Animals.

[15] Zhang X., Zhang S., Tang Q., Jiang E., Wang K., Lan X., Pan C. (2020). Goat sperm associated antigen 17 protein gene (SPAG17): Small and large fragment genetic variation detection, association analysis, and mRNA expression in gonads. Genomics.

[16] Yang L., Niu Q., Zhang T., Zhao G., Zhu B., Chen Y., Zhang L., Gao X., Gao H., Liu G.E. (2021). Genomic sequencing analysis reveals copy number variations and their associations with economically important traits in beef cattle. Genomics.

[17] Sebat J., Lakshmi B., Troge J., Alexander J., Young J., Lundin P., Månér S., Massa H., Walker M., Chi M. (2004). Large-scale copy number polymorphism in the human genome. Science.

[18] Liu M., Woodward-Greene J., Kang X., Pan M.G., Rosen B., Van Tassell C.P., Chen H., Liu G.E. (2020). Genome-wide CNV analysis revealed variants associated with growth traits in African indigenous goats. Genomics.

[19] Kang X., Li M., Liu M., Liu S., Pan M.G., Wiggans G.R., Rosen B.D., Liu G.E. (2020). Copy number variation analysis reveals variants associated with milk production traits in dairy goats. Genomics.

[20] Li L., Yang P., Shi S., Zhang Z., Shi Q., Xu J., He H., Lei C., Wang E., Chen H. (2020). Association analysis to copy number variation (CNV) of Opn4 gene with growth traits of goats. Animals.

[21] Wang Z., Wang C., Guo Y., She S., Wang B., Jiang Y., Bai Y., Song X., Li L., Shi L. (2020). Screening of deletion variants within the goat PRDM6 gene and its effects on growth traits. Animals.

[22] Xu Z., Wang X., Zhang Z., An Q., Wen Y., Wang D., Liu X., Li Z., Lyu S., Li L. (2020). Copy number variation of CADM2 gene revealed its association with growth traits across Chinese Capra hircus (goat) populations. Gene.

[23] Bi Y., Feng W., Kang Y., Wang K., Yang Y., Qu L., Chen H., Lan X., Pan C. (2021). Detection of mRNA expression and copy number variations within the goat Fec^B^ gene associated with litter size. Front. Vet. Sci..

[24] Wang K., Kang Z., Jiang E., Yan H., Zhu H., Liu J., Qu L., Lan X., Pan C. (2020). Genetic effects of DSCAML1 identified in genome-wide association study revealing strong associations with litter size and semen quality in goat (Capra hircus). Theriogenology.

[25] Hui Y., Zhang Y., Wang K., Pan C., Chen H., Qu L., Song X., Lan X. (2020). Goat DNMT3B: An indel mutation detection, association analysis with litter size and mRNA expression in gonads. Theriogenology.

[26] Gangwar C., Mishra A.K., Gururaj K., Kumar A., Kharche S.D., Saraswat S., Kumar R., Ramachandran N. (2021). Semen quality and total microbial load: An association study in important Indian Goat breeds during different seasons. Andrologia.

[27] Campbell R.C., Dott H.M., Glover T.D. (1956). Nigrosin eosin as a stain for differentiating live and dead spermatozoa. J. Agric. Sci..

[28] Abril-Sánchez S., Crosignani N., Freitas-de-Melo A., Terrazas A., Damián J.P., Beracochea F., Silveira P., Ungerfeld R. (2018). Sedation or anaesthesia decrease the stress response to electroejaculation and improve the quality of the collected semen in goat bucks. Animal.

[29] Jeyendran R.S., Van der Ven H.H., Perez-Pelaez M., Crabo B.G., Zaneveld L.J. (1984). Development of an assay to assess the functional integrity of the human sperm membrane and its relationship to other semen characteristics. J. Reprod. Fertil..

[30] Li R., Fu W., Su R., Tian X., Du D., Zhao Y., Zheng Z., Chen Q., Gao S., Cai Y. (2019). Towards the complete goat pan-genome by recovering missing genomic segments from the reference genome. Front. Genet..

[31] Shi S.Y., Li L.J., Zhang Z.J., Wang E.Y., Wang J., Xu J.W., Liu H.B., Wen Y.F., He H., Lei C.Z. (2020). Copy number variation of MYLK4 gene and its growth traits of Capra hircus (goat). Anim. Biotechnol..

[32] Wang X., Zheng Z., Cai Y., Chen T., Li C., Fu W., Jiang Y. (2017). CNVcaller: Highly efficient and widely applicable software for detecting copy number variations in large populations. Gigascience.

[33] Huang Y., Su P., Akhatayeva Z., Pan C., Zhang Q., Lan X. (2022). Novel InDel variations of the Cry2 gene are associated with litter size in Australian White sheep. Theriogenology.

[34] Kang Z., Bai Y., Lan X., Zhao H. (2021). Goat AKAP12: Indel mutation detection, association analysis with litter size and alternative splicing variant expression. Front. Genet..

[35] Bai Y., Yuan R., Luo Y., Kang Z., Zhu H., Qu L., Lan X., Song X. (2021). Exploration of genetic variants within the goat A-Kinase anchoring protein 12 (AKAP12) gene and their effects on growth traits. Animals.

[36] Miyata H., Satouh Y., Mashiko D., Muto M., Nozawa K., Shiba K., Fujihara Y., Isotani A., Inaba K., Ikawa M. (2015). Sperm calcineurin inhibition prevents mouse fertility with implications for male contraceptive. Science.

[37] Rusnak F., Mertz P. (2000). (2000). Calcineurin: Form and function. Physiol. Rev..

[38] Dey S., Eisa A., Kline D., Wagner F.F., Abeysirigunawardena S., Vijayaraghavan S. (2020). Roles of glycogen synthase kinase 3 alpha and calcineurin in regulating the ability of sperm to fertilize eggs. FASEB J..

[39] Akhatayeva Z., Mao C., Jiang F., Pan C., Lin C., Hao K., Lan T., Chen H., Zhang Q., Lan X. (2020). Indel variants within the PRL and GHR genes associated with sheep litter size. Reprod. Domest. Anim..

[40] Kang Z., Jiang E., Wang K., Pan C., Chen H., Yan H., Zhu H., Liu J., Qu L., Lan X. (2019). Goat membrane associated ring-CH-type finger 1 (MARCH1) mRNA expression and association with litter size. Theriogenology.

